# Epigenetic inactivation of DNA repair genes as promising prognostic and predictive biomarkers in urothelial bladder carcinoma patients

**DOI:** 10.1007/s00438-022-01950-x

**Published:** 2022-09-08

**Authors:** Marwa Mohanad, Hend F. Yousef, Abeer A. Bahnassy

**Affiliations:** 1 Biochemistry Department, College of Pharmaceutical Sciences and Drug Manufacturing, Misr University for Science and Technology, 6th of October, Giza, Egypt; 2 Tissue Culture and Cytogenetics Unit, Pathology Department, National Cancer Institute, Cairo University, Cairo, Egypt

**Keywords:** CtIP/RBBP8, MSH4, Urothelial bladder carcinoma, Epigenetic inactivation, Machine-learning predictions of response, Prognosis

## Abstract

We sought to examine epigenetic inactivation of DNA damage repair (DDR) genes as prognostic and predictive biomarkers for urothelial bladder cancer (UBC) as there are currently no reliable prognostic biomarkers that identify UBC patients who would benefit from chemotherapy. Genome-wide DNA methylome using the cancer genome atlas-bladder cancer (TCGA-BLCA) datasets (primary tumors = 374 and normal tissues = 37) was performed for 154 DDR genes. The most two significant differentially methylated genes, Retinoblastoma binding protein 8 (*RBBP8)* and MutS homologue 4 *(MSH4)*, between primary tumors and normal tissues of TCGA–BLCA were validated by methylation-specific PCR (MSP) in UBC (*n* = 70) compared to normal tissues (*n* = 30). *RBBP8* and *MSH4* expression was measured using qRT-PCR. We developed a predictive model for therapeutic response based on the *RBBP8*- and *MSH4*-methylation along with patients’ clinical features*.* Then, we assessed the prognostic significance of *RBBP8* and *MSH4*. *RBBP8-* and *MSH4* methylation and corresponding gene downregulation significantly associated with muscle-invasive phenotype, prolonged progression-free survival (PFS) and increased susceptibility to cisplatin chemotherapy in UBC. Promoter methylation of *RBBP8* and *MSH4* was positively correlated with each other and with their corresponding gene repression. The best machine-learning classification model predicted UBC patients’ response to cisplatin-based chemotherapy with an accuracy of 90.05 ± 4.5%. Epigenetic inactivation of *RBBP8* and *MSH4* in UBC could sensitize patients to DNA-damaging agents. A predictive machine-learning modeling approach based on the clinical features along with *RBBP8-* and *MSH4*-methylation might be a promising tool for stratification of UBC responders from nonresponders to chemotherapy.

**Supplementary Information:**

The online version contains supplementary material available at 10.1007/s00438-022-01950-x.

## Introduction

Bladder cancer (BLCA) is the most prevalent malignancy of the urinary tract and has the highest recurrence rate ranging from 50 to 90% (Siegel et al. 2019). Urothelial bladder carcinoma (UBC) accounts for 94% of bladder cancer cases and can be categorized as either muscle-invasive urothelial bladder carcinoma (pT2, pT3, or pT4; MIBC) or nonmuscle-invasive urothelial bladder carcinoma (pTa or pT1; NMIBC). The majority of NMIBC are associated with high risk of recurrence and progression to MIBC (Halperin et al. 2019). Cancer-specific survival in patients with MIBC is unfavorable despite treatments with radical cystectomy with or without perioperative cisplatin chemotherapy (Alfred Witjes et al. 2017). Thus, there is a need for novel prognostic and predictive biomarkers that will aid in identifying high-risk UBC patients who may benefit from chemotherapy. UBC is a heterogenous disease that is associated with genetic and epigenetic instability that drive the progression and aggressiveness of cancer (Martinez et al. 2019).

Epigenetic changes chiefly differential DNA methylation (DNAm) pattern represents the major form of epigenetic modifications that control gene expression early in carcinogenesis and holds promise as prognostic biomarker for cancer due of its well-recognized association with various aspects of human cancer (Alvarez et al. 2011; Patil and Herceg 2019). It has been reported that aberrant promoter methylation of DNA damage repair (DDR) genes play a crucial role in cancer risk diagnosis, prognosis and stratification of patients with distinct risk of treatment response (Magzoub et al. 2019).

The identification of aberrantly methylated and differentially expressed genes might provide potential epigenetic biomarkers for UBC. In this study, we evaluated differential DNAm levels of 154 DDR genes using the cancer genome atlas (TCGA)–BLCA DNA–methylome data. Consequently, we aimed to investigate the prognostic value of the most significant differentially methylated genes *RBBP8* and *MSH4* in an institutional cohort of UBC patients and to develop classification model for prediction of pathological response to therapy in UBC patients based on the *RBBP8* and *MSH4* methylation.

Retinoblastoma binding protein 8 (*RBBP8*), also known as C-terminal binding protein (CtBP)-interacting protein (CtIP) encodes extensively expressed nuclear endonuclease. Accumulating studies have reported that RBBP8 is required for DNA double-stranded break (DSB) repair by homologous recombination (HR) in G2/M phases through interaction with BRCA1 and MRE11-RAD50-NBN (MRN) complex (Huertas and Jackson 2009; Sartori et al. 2007). RBBP8 interacts with tumor suppressor genes such as BRCA1 and the pRb family members through binding domains that are frequently mutated in human cancers (Chinnadurai 2006). Some studies suggested that disruption of BRCA1–RBBP8 interaction results in cell cycle arrest modulation (Li et al. 2000; Wu-Baer and Baer 2001). Mismatch repair (MMR) genes play a crucial role in DNA repair mechanism. Loss of function of MMR genes by mutation, loss of heterozygosity or promoter hypermethylation affect its role in repairing intranucleotide error (Spetsotaki et al. 2017). MutS homolog (*MSH)4* plays a crucial role in maintaining genomic stability through nonhomologous end joining (NHEJ) pathway to DSB (Chu et al. 2013). It has been reported that a single nucleotide polymorphism (SNP)–SNP interaction between *MSH4* Ala97Thr/*MLH3* Leu844Pro increases breast cancer susceptibility (Conde et al. 2009). Promoter hypermethylation and downregulation of *MSH4* have also been shown in head and neck squamous cell carcinoma. However, the role of aberrant *MSH4* expression have not been previously reported in bladder tumors (Chaisaingmongkol et al. 2012). In this study, we demonstrate for the first time the prognostic and predictive role of *RBBP8 and MSH4* hypermethylation for UBC disease.

## Subjects and methods

### Study population

The study protocol was approved by institutional Review Board (IRB) of National Cancer Institute (NCI), Cairo, Egypt—as guided by the 2013 Helsinki Declaration (IRB NO.IRB00001568). All subjects provided signed informed consent for collection and analysis of their specimens. Patients with history of other malignancy and carcinoma in situ were excluded from the study. A total of 70 formalin fixed paraffin embedded (FFPE) bladder tissues of patients undergoing radical cystectomy were recruited from NCI, Egypt during the period from January 2016 to October 2018. 30 adjacently normal urothelium were included as normal controls (NC). The lack of significant inflammation or atypia confirm the diagnosis of normal tissues. Follow-up data were acquired prospectively from clinic visits and electronic patient records. All patients received adjuvant and/or neoadjuvant cisplatin-based chemotherapy. Response to treatment was assessed based on the response evaluation criteria in solid tumors (RECIST) (Schwartz et al. 2016). For data analysis complete and partial response were grouped into responders while, stable and progressive disease were grouped into nonresponders.

### In silico analyses

The DNAm, gene expression (RNA-seq) and the corresponding clinical data of TCGA−BLCA (primary tumors = 397 and normal tissues = 37) (https://portal.gdc.cancer.gov/) were downloaded and assessed by TCGA-assembler 2 (Wei et al. 2018). Briefly, after data download, we performed advanced processing to retrieve average DNA methylation values (B value) of CpG sites in a specific gene location (e.g. the promoter region) and mRNA expression in transcript per million (TPM). We determined the average methylation level for each of 154 DDR genes within the gene promoter region generated by KEGG database searches for DNA damage repair, MMR, NHEJ, HR, DDR checkpoint, base excision repair (BER), and nucleotide excision repair (NER) (Supplementary Table 1). The criteria for screening of significant differentially methylated genes were Beta value > 0.2 and corrected *p* value < 0.05 (independent *t* test plus Benjamini–Hochberg method). The differential expression of *RBBP8 *and *MSH4 *genes in bladder cancer samples compared to normal control were confirmed by GEO13507 dataset using GEOquery and limma package of R studio. Ensembl biomart provided all the necessary genomic DNA information required to identify *RBBP8* and *MSH4* gene core promoter region (ENSG00000101773 and ENSG00000057468, respectively) (https://m.ensembl.org/biomart/) that can be used to design the required methylation-specific PCR (MSP) primers. Consequently, refTSS (http://reftss.clst.riken.jp/r) provided CpG island locations (22,933,156–22,933,894) on chromosome 18 at  – 663 to + 75 corresponding to transcription start site (TSS: 22,933,819) of *RBBP8* and 75,796,851–75,797,218 on chromosome 1 at -34 to + 333 corresponding to TSS: 75,796,885. The selected CpG-rich island fulfilled the following conditions: GC content ≥ 50%, ratio Obs/Exp CpG dinucleotide ≥ 0.6 and the length of genomic region > 200 bp ( http://dbcat.cgm.ntu.edu.tw/).

### Total DNA and RNA extraction

Sections of FFPE tissue blocks were deparaffinized by xylene and rehydrated prior to nucleic acid extraction. Genomic DNA and RNA were extracted from FFPE tissues using Genedirex DNA extraction kit for tissue (GENEDIREX, INC, Taiwan, China) and Genedirex total RNA extraction kit (GENEDIREX, INC, Taiwan, China), respectively, according to manufacturer’s instructions.

Nucleic acid extraction for UBC tissues was performed in histologically confirmed areas containing a minimum of 70% tumor cells. Nucleic acid quality and concentration were assessed using NanoDrop 2000 (Thermoscientific, USA) with A260/A230 for DNA and A260/A280 ratio for RNA between 1.8 and 2.2.

### Bisulfite conversion and methylation-specific PCR (MSP)

DNA methylation of *RBBP8* and *MSH4* was determined by bisulfite conversion of unmethylated cytosines to uracil using methylation-specific PCR (MSP) (Huang et al. 2013). In brief, 100 to 400 ng of the extracted DNA was subjected to bisulfite conversion using the EpiJET Bisulfite Conversion Kit (ThermoFisher Scientific, USA) according to the manufacturer's protocol followed by amplification of 150–300 ng of the bisulfite-treated DNA a set of MSP primers (Supplementary Table 2). All PCR reactions were performed by the Veriti Thermo Cycler (Life Technologies, Carlsbad, CA, USA). The MSP products were separated on 2% agarose gels, stained with ethidium bromide, and visualized using UV transilluminator.

### Quantitative real time PCR (qRT-PCR)

An one-step qRT-PCR was accomplished using SYBR® Green RT-qPCR Master Mix (Willowfort.co/UK) including 5 μM of oligonucleotide primers (Supplementary Table 3) and 150 ng of extracted RNA. All reactions were done in triplicates using 7500 Fast-Real time PCR system software (Applied Biosystem, USA) and postamplification curves was assessed for product specificity. The fold change (FC) expression was calculated relative to glyceraldehyde 3-phosphate dehydrogenase *(GAPDH*) housekeeping gene by 2^−ΔΔCt^ method (Bahnassy et al. 2019).

### Prediction model

We used Python sklearn library to develop predictive model for response to therapy based on the most relevant nonredundant patients’ characteristics along with *RBBP8* and *MSH4* methylation data as shown in Supplementary Fig. 1. The most relevant nonredundant clinical characteristics were selected using the rank ordering method of the SelectKBest class of python scikit-learn library. We tested different classification models (logistic regression model (LR), kernel support vector machine (SVM), K-nearest neighbor (KNN), Decision tree (DT) and Random Forrest decision tree (RF)) for the best prediction of treatment response. sklearn voting ensemble was used to find the best model’s combination. Initially dataset was split into 80% training-set and 20% test-set. Grid-search method was used to optimize each classifier respective hyperparameters and a10-fold cross validation on the training-set was used to evaluate the model on an independent validation sets to avoid model overfitting. Performance of each classifier was measured by its accuracy and area under the Receiver operating characteristic (ROC) curve (AUC).

### Statistical analysis

All statistical analysis was performed using R studio Statistical Software (version 3.7, Vienna, Austria). The pwr package was used to adjust the power of the test. Comparison of differential expression between study groups and with patients’ clinicopathological features was done using Wilcoxon rank test. Chi-square was used to investigate methylation in association with clinicopathological features and logistic regression was used to estimate association of different parameters with response. The multiple comparisons were adjusted for false discovery rate (FDR) (Benjamini and Hochberg 1995). Pearson correlation was used to measure the correlation coefficient. Progression-free survival (PFS) was calculated from the date of primary therapy to either recurrent or progressive disease, patients free of progressive disease were censored at the time of the last follow up. Kaplan–Meier survival analysis–log-rank test was used to compare survival time. Cox proportional hazard regression analysis were applied to evaluate the hazard of *RBBP8* and *MSH4* along with clinicopathological data on survival probability. Hierarchical clustering heatmap was used to show the methylation values of *DDR* genes based on Infinium HumanMethylation450 BeadChip. All tests were two sides and significance was set at *p* < 0.05.

## Results

### Clinicopathological features

The clinicopathological variable of UBC and NC are shown in Table 1. The mean age of UBC patients and NC was 62.2 ± 8.5 years and 61.9 ± 8.9, respectively; *p* = 1.0).

**Table 1:** Clinicopathological characteristics in urothelial bladder carcinoma (UBC) and normal controls (NC) providing tissue samples

Characteristics	UBC cases (*n* = 70)		NC (*n* = 30)		*p* value
	*n*	%	*n*	%	
Age (yrs)	62.2 ± 8.0		61.9 ± 8.9		1.0^a^
*Age (yrs)*					
< 62	28	40.0	14	46.7	0.69^b^
≥ 62	42	60.0	16	53.3	
*Gender*					
Female	11	15.7	10	33.3	0.08^b^
Male	59	84.2	20	66.7	
*Smoking*					
Nonsmoker	44	62.9	22	73.3	0.43^b^
Smoker	26	37.1	8	26.7	
*Tumor size(cm)*	4.3 ± 2.1				
*Tumor size(cm)*					
< 4	32	45.7			
≥ 4	38	54.3			
*Grade*					
Low (1–2)	38	54.3			
High (3)	32	45.7			
*Stage*					
Early (0, I, II)	30	42.9			
Late (III−IV)	40	57.1			
*Type*					
NMIBC	25	35.7			
MIBC	45	64.3			
*LN metastasis*					
No	57	81.4			
Yes	13	18.6			

*LN* lymph node, *NMIUC* nonmuscle-invasive urothelial bladder cancer, *MIBC* muscle-invasive urothelial bladder cancer

^a^Wilcoxon rank test. ^b^Chi square test

### Genome-wide DNA methylation pattern

**Fig. 1:**
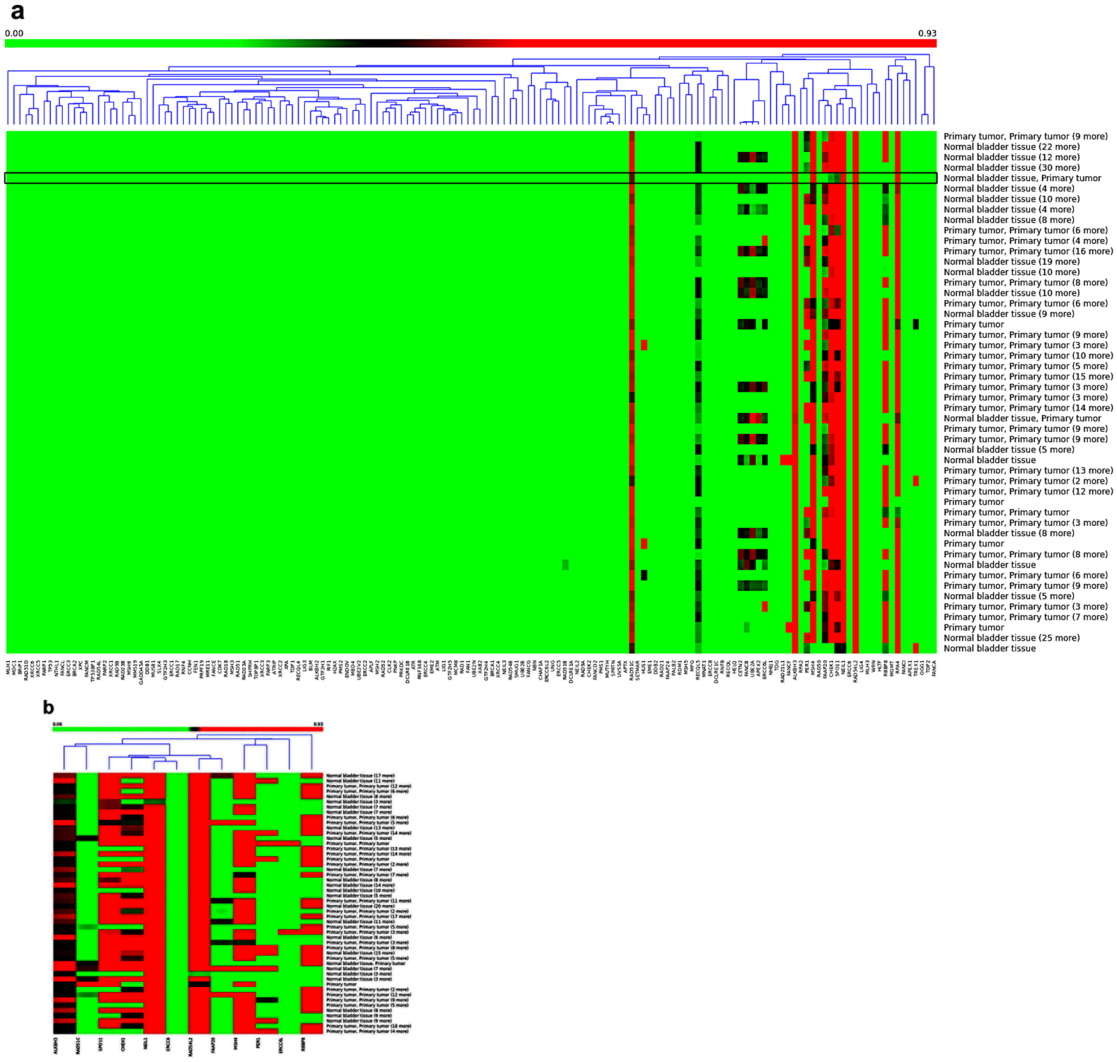
Hierarchical clustered Heatmap. **a** DNA damage repair (DDR) genes using DNA methylation data in TCGA−BLCA dataset. **b** Significantly hypermethylated DDR genes (B value > 0.2) in TCGA−BLCA dataset. Red boxes represent high methylated genes while green boxes indicate low methylated genes. The column represents individual DDR genes while rows represent TCGA−BLCA primary tumor and normal tissue samples

**Fig. 2:**
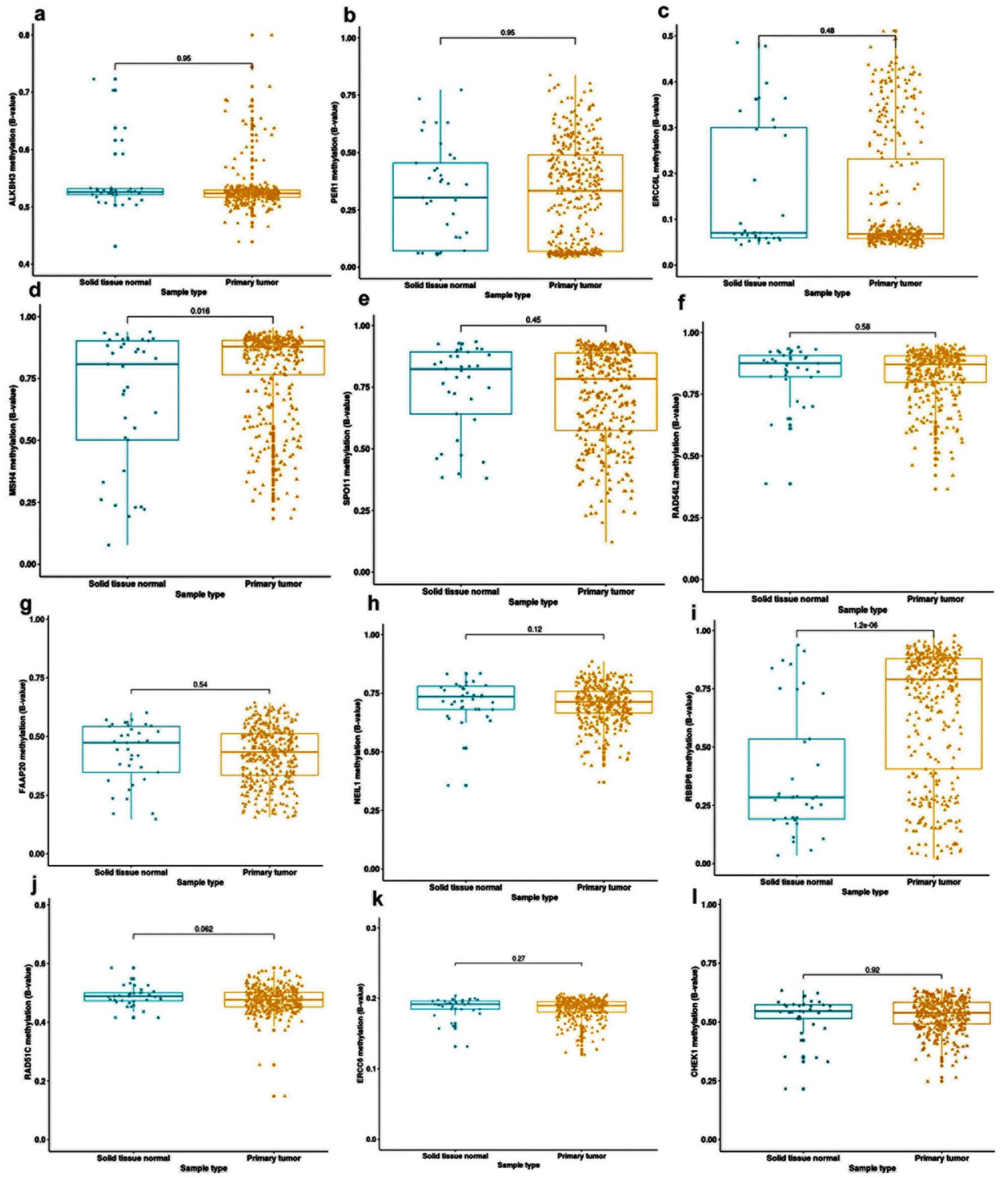
Differential methylation of the 12 hypermethylated DDR genes (B value > 0.2) between primary tumor and normal tissues of TCGA−BLCA dataset. **a** *ALKBH3*, **b** *PER1*, **c** *ERCCL6*, **d** *MSH4*, **e** *SPO11*, **f** *RAD54L2*, **g** *FAAP20*, **h** *NEIL1*, **i** *RBBP8*, **j** *RAD51C*, **k** *ERCC6*, and **l** *CHEK1*

We investigated the methylation pattern of 154 DDR genes using TCGA−BLCA datasets. Hierarchical clustering showed that 12 genes (*ALKBH3*, *PER1*, *ERCC6L, MSH4, SPO11, RAD54L2, FAAP20, NEIL1, RBBP8, RAD51C, ERCC6* and *CHEK1*) were hypermethylated in TCGA−BLCA datasets (average B values > 0.2) (Fig. 1). Then, we compared the differential methylation of the 12 hypermethylated DDR genes between 374 primary bladder tumors and 37 normal bladder tissues as shown in Fig. 2. We identified that *RBBP8* and *MSH4* were the most significantly hypermethylated genes in bladder tumors than in normal tissues (*p* < 0.001 and *p* = 0.016, respectively) (Fig. 2d, i).

### Validation of *RBBP8* and *MSH4* differential methylation between UC and NC

**Fig. 3:**
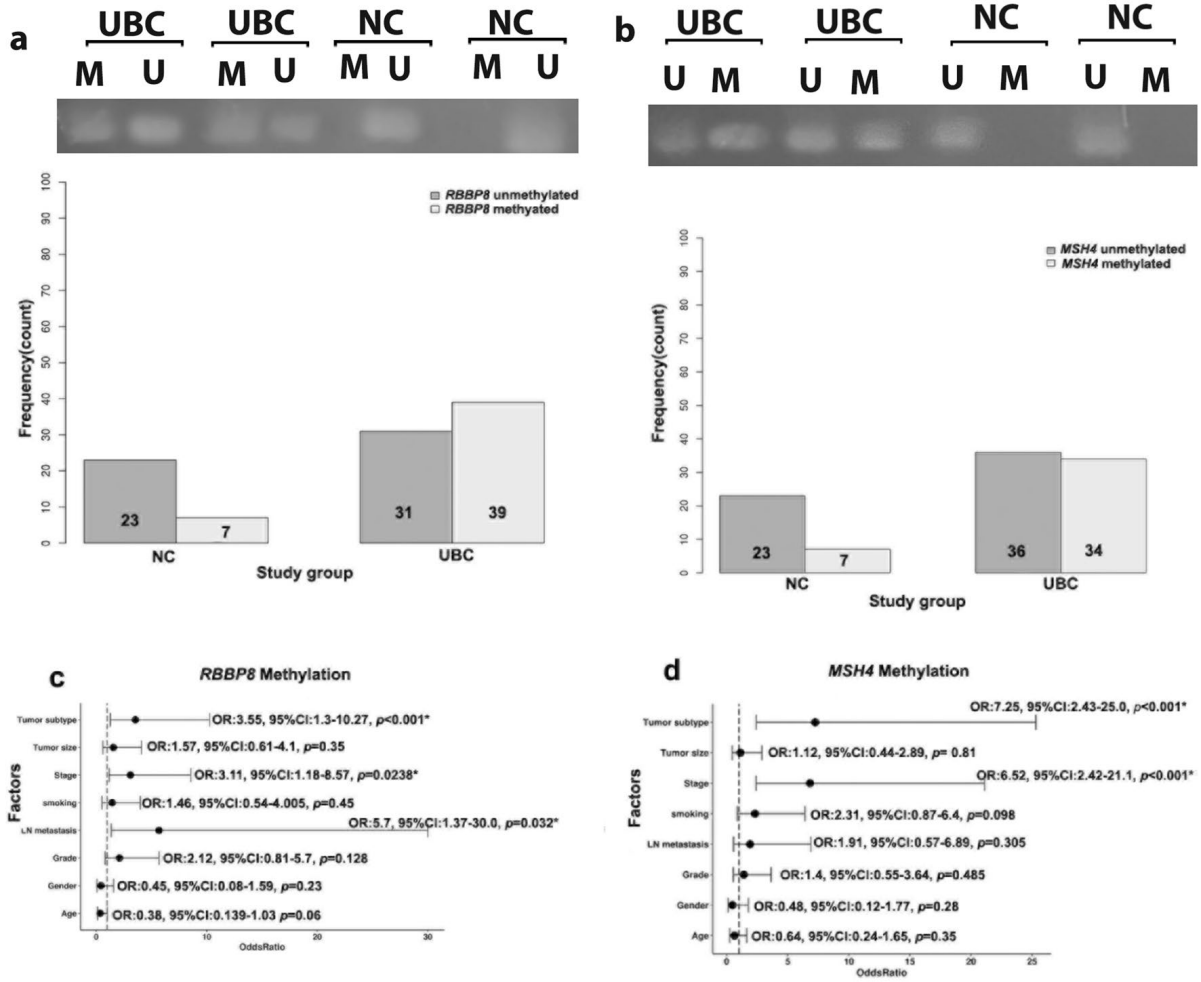
*RBBP8* and *MSH4* methylation in study groups (amplicon size of 121 bp and 263 bp, respectively). Representative of MSP results of **a** *RBBP8* methylation in UBC and NC tissues, and bar plot of frequency of *RBBP8* expression among UBC and NC. **b** *MSH4* methylation in UBC and NC tissues, and bar plot of frequency of *MSH4* expression among UBC and NC samples of urothelial carcinoma patients and normal controls. **c** Forest plot showing the odds for *RBBP8* methylation in association with clinicopathological features. **d** Forest plot showing the odds for *MSH4* methylation in association with clinicopathological features. *NC* normal tissue, *UBC* urothelial bladder carcinoma tissue

Using MSP in our cohort (UBC = 70 and NC = 30) as shown in Fig. 3, we found that the frequency of *RBBP8* and *MSH4* methylation was significantly higher in UBC tissues (39/70, 55.7% and 34/70, 48.57%, respectively) compared to NC (7/30, 23.3% and 7/30, 23.3%, respectively) (*p* = 0.003 and *p* < 0.001, respectively).

### Association of *RBBP8 *and *MSH4*-methylation with patients’ characteristics

As shown in Table 2, *RBBP8* methylation was significantly associated with late stage (67.5%, *p* = 0.0238), muscle-invasive disease (66.7%, *p* < 0.001) and LN metastasis (84.6%, *p* = 0.032) as compared to *RBBP8* unmethylation (32.5%, 33.3% and 15.4%, respectively). *MSH4* methylation was significantly associated with late stage (67.5%, *p* < 0.001) and muscle-invasive disease (64.4%, *p* < 0.001) as compared with *MSH4* unmethylation (32.5% and 35.6%, respectively). Using logistic regression, we found that odds *RBBP8* methylation significantly increased with late stage [OR: 3.11, 95% CI 1.18–8.57, *p* = 0.0238], LN metastasis (OR: 5.7, 95% CI 1.37–30, *p* = 0.032) and muscle-invasive disease (OR: 3.55, 95% CI 1.3–10.27, *p* < 0.001) (Fig. 3c). For *MSH4,* methylation odds significantly increased with late tumor stage [OR: 6.82, 95% CI 2.42–21.1, *p* < 0.001] and muscle-invasive disease [OR: 7.25, 95% CI 2.43–25.0, *p* < 0.001] (Fig. 3d).

**Table 2:** Frequency of *RBBP8* and *MSH4* Methylation in relation to clinicopathological features

	*n*	*RBBP8* N (%)		*p* value	*MSH4 N* (%)		*p* value
		UM	M		UM	M	
*Age*							
< 60	29	9(31.03)	20(68.96)	0.06	13(44.8)	16(55.2)	0.35
≥ 60	41	22(53.7)	19(46.3)		23(56.1)	18(43.9)	
*Gender*							
Female	11	3(27.3)	8(72.7)	0.23	4(36.4)	7(63.6)	0.28
Male	59	28(47.5)	31(52.5)		32(54.2)	27(45.8)	
*Tumor size*							
< 4	34	17(50.0)	17(50.0)	0.35	18(52.9	16(47.1)	0.81
≥ 4	36	14(45.2)	22(56.4)		18(50.0)	18(50.0)	
*Grade*							
Low(G1-2)	38	20(52.6)	18(47.4)	0.128	21(55.3)	17(44.7)	0.485
High (G3)	32	11(34.4)	21(65.6)		15(46.9)	17(53.1)	
*Stage*							
Early(0–II)	30	18(6.0)	12(40.0)	0.0238*	23(76.7)	7(23.3)	< 0.001*
Late (III–IV)	40	13(32.5)	27(67.5)		13(32.5)	27(67.5)	
*Tumor subtype*							
NMIBC (pTa-T1)	25	16(64.0)	9(36.0)	< 0.001*	20(80.0)	5(20.0)	< 0.001*
MIBC (pT2-T4)	45	15(33.3)	30(66.7)		16(35.6)	29(64.4)	
*LN metastasis*							
No	57	29(50.9)	28(49.1)	0.032*	31(54.4)	26(45.6)	0.305
Yes	13	2(15.4)	11(84.6)		5(38.5)	8(61.5)	
*Smoking*							
No	44	21(47.7)	23(52.3)	0.45	26(59.1)	18(40.9)	0.098
Yes	26	10(38.5)	16(61.5)		10(38.5)	16(61.5)	

*RBBP8* retinoblastoma binding protein 8, *MSH4* MutS homologue 4, *LN* lymph node, *NMIBC* nonmuscle-invasive urothelial bladder carcinoma, *MIBC* muscle-invasive urothelial bladder carcinoma. *Significant at *p* < 0.05. The data were compared using chi-square test

### Differential expression of RBBP8 and MSH4 mRNA in TGCA and GEO datasets

**Fig. 4:**
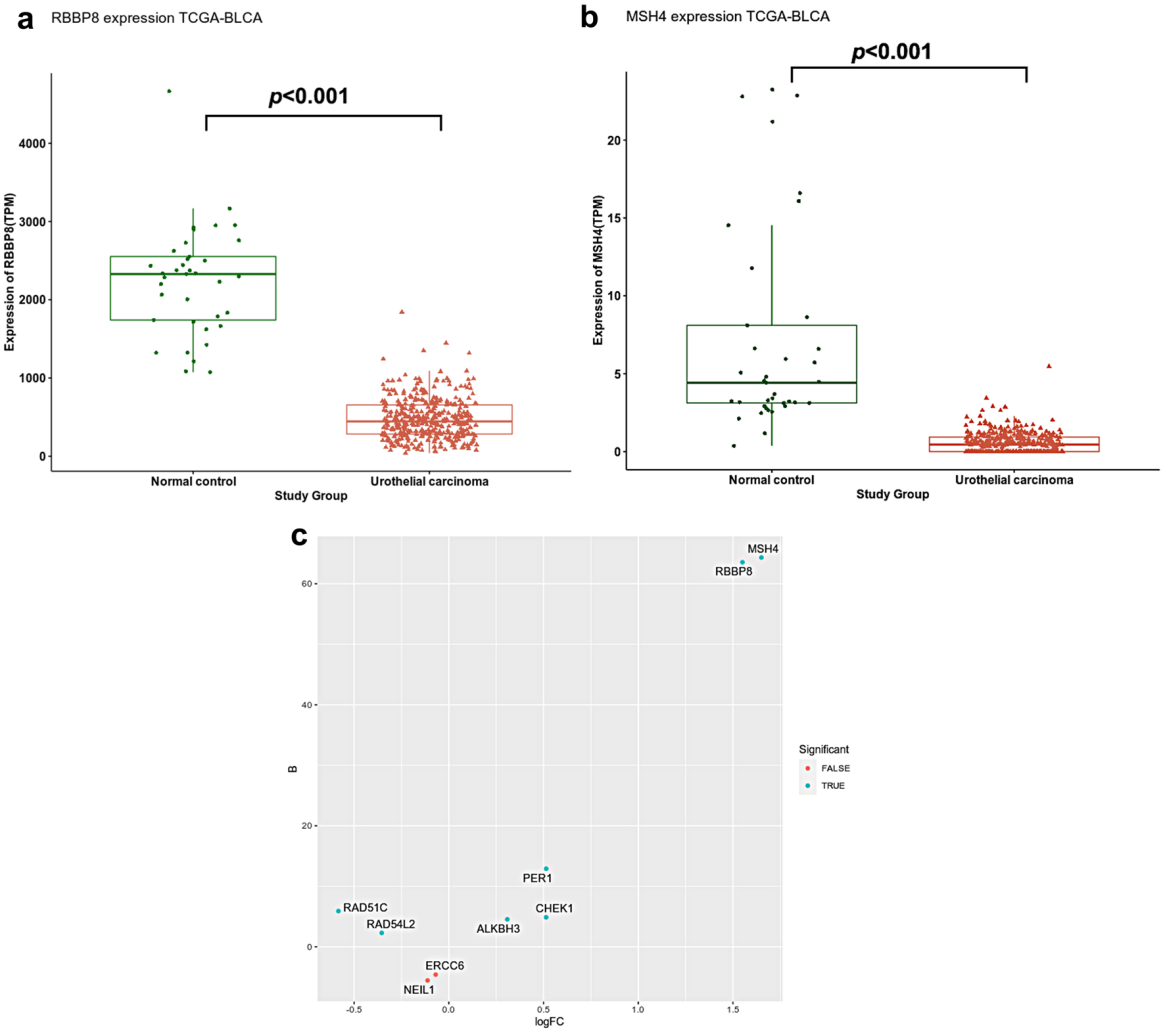
Differential *RBBP8* and *MSH4* expression between UC and normal bladder tissues. **a** Boxplot showing differential *RBBP8* expression between urothelial carcinoma and normal controls in TCGA−BLCA dataset. **b** Boxplot showing differential *MSH4* expression between urothelial carcinoma and normal controls in TCGA−BLCA dataset. **c** Volcano plot for differential expression of the significant differentially methylated genes between UC and normal control. The *x* axis shows the log-fold change, and the *y* axis is some measure of the B score statistical significance

As shown in Fig. 4, the TCGA−BLCA dataset showed that the expression of *RBBP8 *and *MSH4 *was significantly lower in bladder cancer cases as compared to normal samples (*p* < 0.001). Furthermore, GEO13507 dataset showed that *RBBP8* and *MSH4 *were differentially expressed in bladder cancer cases compared normal tissues by 1.82 and 1.88 fold, respectively (*p* < 0.001).

### Validation of *RBBP8* and *MSH4* differential mRNA expression between UBC and NC

**Fig. 5:**
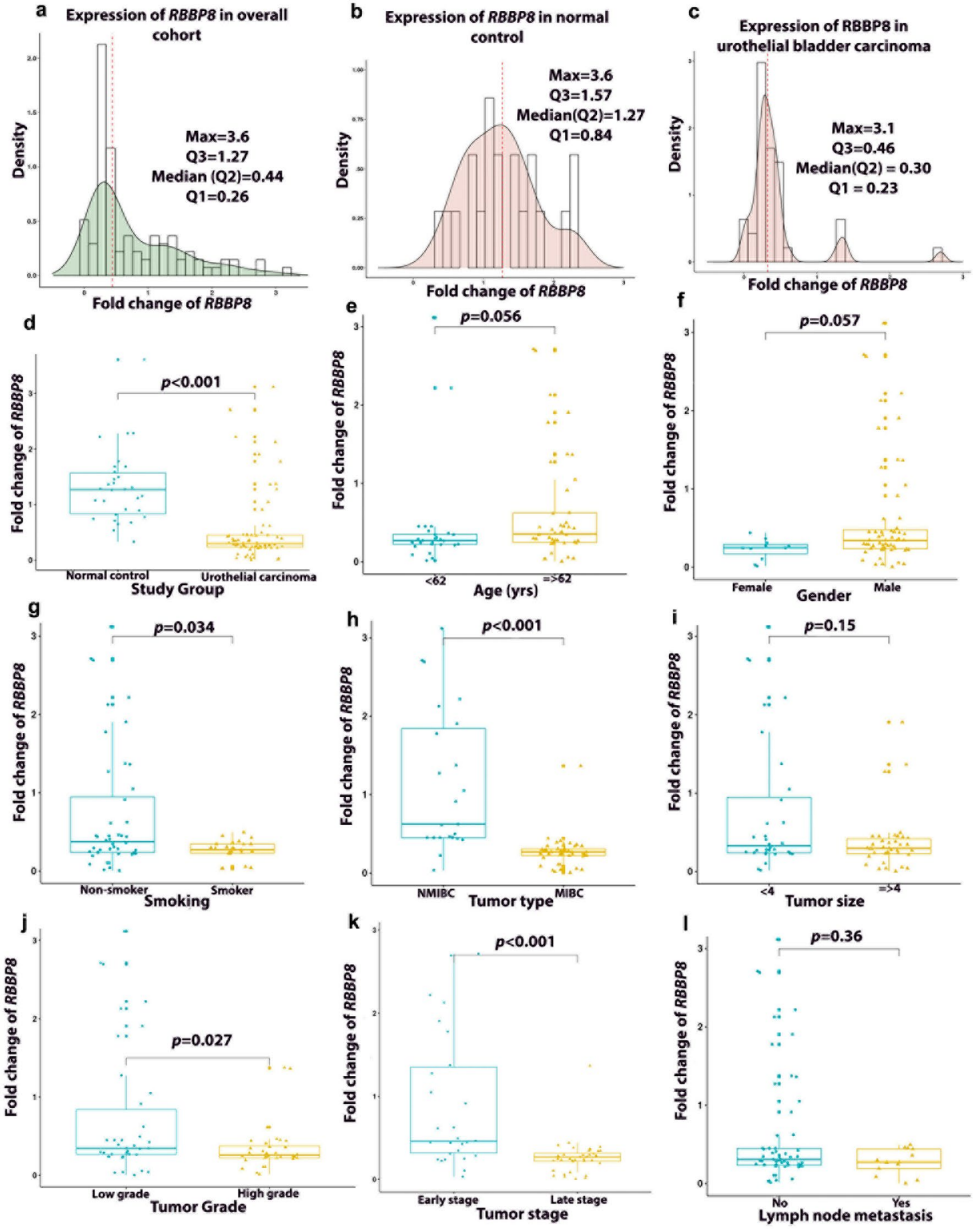
Fold change of *RBBP8* expression. **a** Box plot representing significant increase in *RBBP8* expression in NC compared to UC. Histogram density distribution of *RBBP8* fold change expression, in **b** overall cohort, **c** normal controls and **d** urothelial carcinoma. **e**–**l** Box plot representing association of *RBBP8* expression and UC patients’ characteristics. *NMIBC* nonmuscle invasive urothelial bladder cancer, *MIBC* muscle-invasive urothelial bladder cancer. *Significant at *p* < 0.05. Association between *RBBP8* and clinicopathological features was tested using Wilcoxon sum ran test

**Fig. 6:**
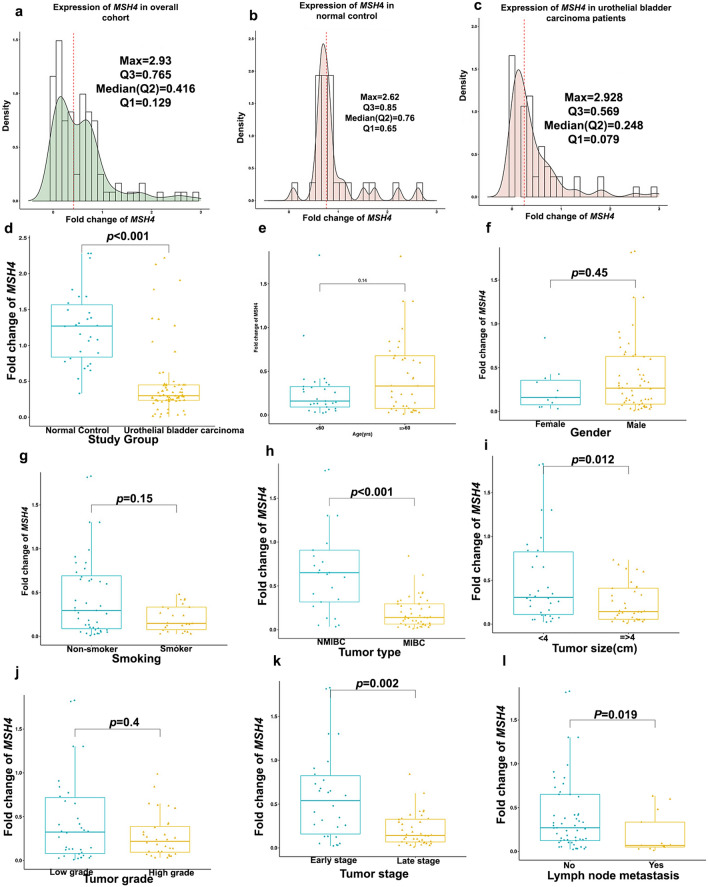
Fold change of *MSH4* expression. (**a**) Box plot representing significant increase in *MSH4* expression in NC compared to UC. Histogram density distribution of *MSH4* fold change expression, in **b** overall cohort, **c** normal controls and **d** urothelial carcinoma. **e**–**l** Box plot representing association of *MSH4* expression and UC patients’ characteristics. *NMIBC* nonmuscle invasive urothelial bladder cancer, *MIBC* muscle-invasive urothelial bladder cancer. *Significant at *p* < 0.05. Association between *MSH4* and clinicopathological features was tested using Wilcoxon sum ran test

As shown in Fig. 5, the median *RBBP8* mRNA FC was significantly lower in UBC patients by 76.4% (*p* < 0.001) as compared to NC. For *MSH4*, median FC was also significantly lower in UBC by 67.4% (*p* < 0.001) as compared to NC (Fig. 6).

### Association of *RBBP8* and *MSH4* mRNA expression with patients’ characteristics

As shown in Fig. 5, median *RBBP8* FC was significantly lower in MIBC tumors (0.27, IQR:0.09) than NMIBC (0.62, IQR:1.33) by 56.5% (*p* < 0.001), high-grade (0.26, IQR:15) than low-grade tumors (0.35, IQR:0.57) by 25.7% (p = 0.027), late stage (0.27, IQR:0.09) than early stage (0.46, IQR: 1.03) by 41.3% (*p* < 0.001) and tumors from nonsmokers (0.27, IQR:11) compared to those of smokers (0.38,IQR:0.71) by 28.95% (*p* = 0.034). As shown in Fig. 6, median *MSH4* FC was significantly lower with LN metastasis (0.069, IQR:0.28) than without LN metastasis (0.27, IQR:0.53) by 74.4% (*p* = 0.019), late stage (0.143, IQR: 0.259) than early stage (0.539, IQR:0.67) by 73.47% (*p* = 0.002), tumor size ≥ 4 cm (0.138, IQR:0.34) than tumor size < 4 cm (0.325, IQR:0.69) by 57.54% (*p* = 0.012) and MIBC (0.139, IQR:0.23) than NMIBC (0.65, IQR:0.59) by 78.61% (*p* < 0.001).

### Correlation of *RBBP8* and *MSH4* methylation and gene expression in UBC

A significant inverse correlation was found between *RBBP8* methylation and its gene expression (*r* =  – 0.66, *p* < 0.001) as well as with *MSH4* (*r* =  – 0.37, *p* < 0.001) expression. *MSH4* methylation showed significant positive correlation with *RBBP8* methylation (*r* = 0.58, *p* < 0.001) while it had a significant negative correlation with *MSH4*- (*r* =  – 0.32, *p* < 0.001) and *RBBP8* expression (*r* =  – 0.57, *p* < 0.001). A moderate positive correlation was also found between *MSH4*- and *RBBP* expression (*r* = 0.50, *p* < 0.001) (Table 3).

**Table 3:** Correlation between expression and methylation pattern of *RBBP8* and *MSH4*

	*RBBP8*	*RBBP8* M	*MSH4*	*MSH4* M
*RBBP8*	–	*r* = – 0.66 *p* < 0.001*	*r* = 0.50 *p* < 0.001*	*r* = – 0.57 *p* < 0.001*
*RBBP8* M	*r* = – 0.66 *p* < 0.001*		*r* = – 0.37 *p* < 0.001*	*r* = 0.58 *p* < 0.001*
*MSH4*	*r* = 0.50 *p* < 0.001*	*r* = – 0.37 *p* < 0.001*	–	*r* = – 0.38 *p* < 0.001*
*MSH4* M	*r* = – 0.57 *p* < 0.001*	*r* = 0.58 *p* < 0.001*	*r* = – 0.32 *p* < 0.001*	–

*r* Correlation coefficient. *Highly significant at *p* < 0.001. Pearson correlation used to calculate correlation coefficient

### Odd ratios of chemotherapy response

Using forward features selection, we selected the most relevant nonredundant clinical features. Table 4 shows the odds of response in association with the relevant nonredundant features as well as *RBBP8* and *MSH4* methylation. *RBBP8* and *MSH4* methylation were significantly associated with increase in response by 57.1% and 65.0%, respectively [OR: 0.429, 95% CI 0.196–0.936*, p* = 0.033 and OR 0.35, 95% CI 0.148–0.827, *p* = 0.017, respectively]. Tumor size (≥ 4 cm) was significantly associated with decrease in response to therapy [OR: 2.38, 95% CI 1.014–5.55, *p* = 0.04].

**Table 4:** Odds ratio of response therapy in association with patients’ clinical data as well as *RBBP8* and *MSH4* methylation and expression

	Odds ratio	95%CI	*p* value
Tumor size	2.38	1.014–5.55	0.04*
Stage	0.78	0.38–1.56	0.48
Grade	1.14	0.82–1.61	0.44
Type	0.85	0.43–1.64	0.61
LN metastatsis	1.20	0.37–4.0	0.76
Smoking	2.0	0.81–4.95	0.134
*RBBP8* M	0.429	0.196–0.936	0.033*
*MSH4* M	0.35	0.148–0.827	0.017*

*CI* confidence interval, *LN* lymph node, *M* methylation. *Significant at *p* < 0.05. Odds ratio was detected using logistic regression

### Predictive machine-learning models for patient’s stratification according to response

**Fig. 7:**
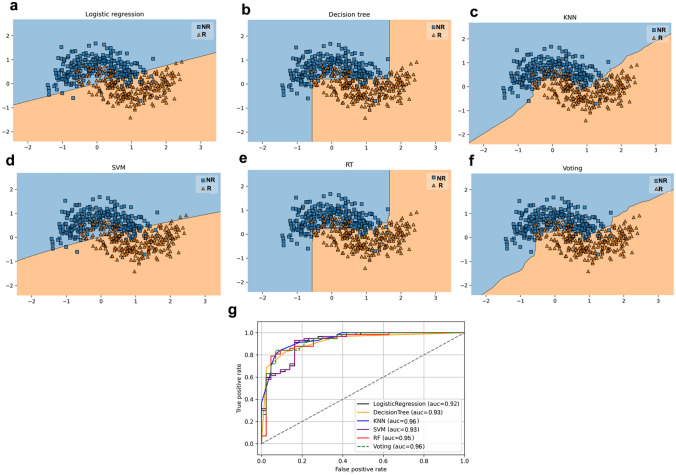
Decision graph showing the distribution of points in feature space based on the performance of model algorithm. **a** Logistic regression, **b** Decision tree, **c** K-nearest neighbor, **d** support vector machine, **e** Random forest tree and **f** voting. **g** Receiver-operating characteristic curve showing AUC for each classifier

We used the rank ordering method of the SelectKBest class of python scikit-learn library to select the most relevant nonredundant features including tumor size, tumor grade, stage, lymph node metastasis. Table 5 displays the performance of classifier with optimum hyperparameter for prediction of UBC patients who respond to chemotherapy based on the *RBBP8* and *MSH4* hypermethylation along with the selected relevant clinical characteristics. The best predictive model was KNN showing an accuracy of 90.05 ± 4.5%, followed by RF having an accuracy of 89.5 ± 3.7%, and DT with an accuracy of 88.5 ± 3.5%. SVM and LR models showed an accuracy of 86.0 ± 4.9% and 85.5 ± 5.2%, respectively. The best model combination was KNN with RF and RT showing an accuracy of 90.0 ± 3.4%, sensitivity of 92.98% and specificity of 81.4%. Figure 7 displays a decision plot for each model that predict the outcome with respect to feature space. The ROC curve showed that KNN, RF, DT, SVM and LR models for prediction of response to therapy in UBC patients had an AUC of 0.96, 0.95, 0.93, 0.93 and 0.92, respectively. The KNN in combination with RF and DT as detected by ensemble voting had an AUC of 0.96.

**Table 5:** Performance of classification model for prediction of response to therapy in for urothelial bladder cancer patients using different machine-learning classifiers

Classifier	Hyperparameter	Accuracy (%)	Sensitivity	Specificity
Logistic regression	Penalty: l2 C: 0.0464	85.5 ± 5.2	82.5	93.03
Decision tree	Best criterion: entropy Best max_depth: 6	88.5 ± 3.4	91.2	83.7
K-nearest neighbor	Algorithm: auto leaf_size: 30, metric: minkowski n_jobs: None n_neighbors: 10	90.0 ± 4.5	87.7	90.7
Support vector machine	Kernel: rbf C: 100 gamma: 0.01	86.0 ± 4.9	92.98	81.4
Random Forest	max_depth: 5 min_samples_leaf: 4 n_estimators: 100	89.5 ± 3.7	87.7	83.7
Voting	KNN + DT + RF	90.0 ± 3.4	92.98	81.4

### Survival analysis

**Fig. 8:**
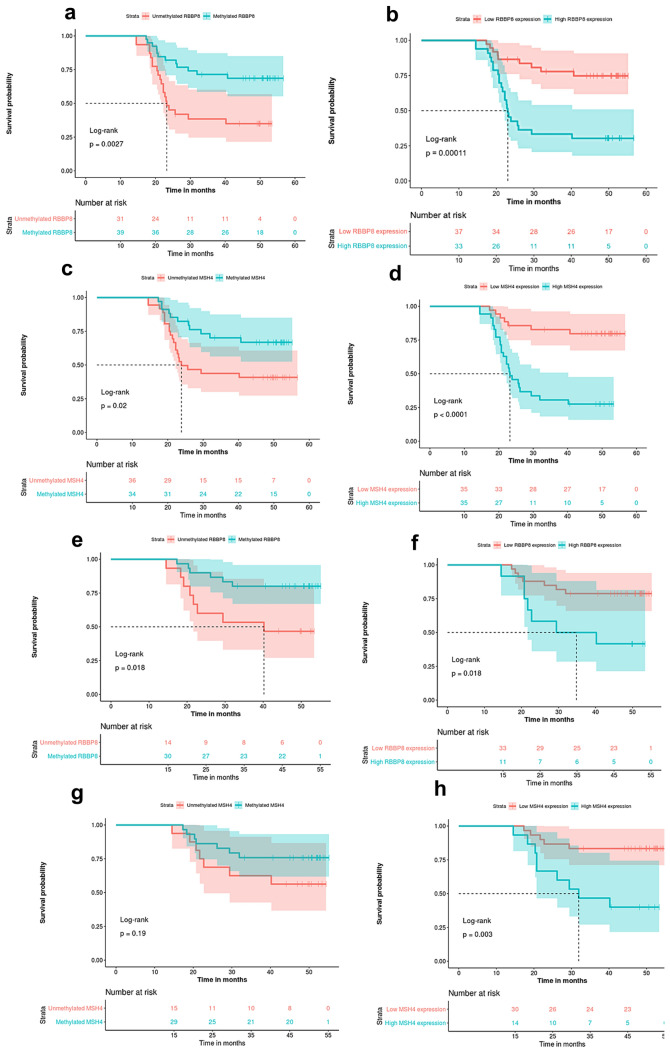
Kaplan–Meier progression-free survival analysis in association with *RBBP8* and *MSH4* methylation and expression. *RBBP8* methylation and expression with PFS in (**a**, **b**) UBC and (**e**, **f**) MIBC. *MSH4* methylation and expression with PFS in (**c**, **d**) UBC and (**g**, **h**) MIBC. Significance at *p* < 0.05. Log-rank test used to analyze survival data

The mean follow-up duration was 48.5 months (range, 14.49–56.67 months). First, the mRNA data of patients were classified into low vs high according to median FC. Kaplan–Meier survival analyses of UBC patients have shown reduced PFS in association with *RBBP8* and *MSH4* methylation (*p* = 0.0027 and *p* = 0.02, respectively, log rank) and reduced expression of corresponding genes (*p* < 0.001, for all, log rank). In MIBC patients, PFS was significantly related to *RBBP8* methylation (*p* = 0.018, log rank) as well as reduced expression of *RBBP8* (*p* = 0.018, log rank) and *MSH4* (*p* = 0.003, log rank) (Fig. 8). In univariate survival analysis, PFS of UBC patients was significantly associated with tumor grade (*p* = 0.006), stage (*p* = 0.007) along with *RBBP8*-M (*p* = 0.029), *MSH4*-M (*p* = 0.023) and their corresponding gene expression (*p* < 0.001 and *p* < 0.001). In MIBC, prolonged PFS was significantly associated with *RBBP8* methylation (*p* = 0.0257) and reduced expression of *RBBP8* and *MSH4* (*p* = 0.007). In multivariate survival-analysis, *MSH4* expression was the only independent prognostic factor for PFS in MIBC patients (*p* = 0.012) on cisplatin-based chemotherapy (Table 6).

**Table 6:** Univariate and multivariate Cox regression survival analysis in overall urothelial bladder carcinoma (*n* = 70) and in muscle-invasive urothelial bladder carcinoma patients (*n* = 45)

Factors	Progression-free survival in UBC			Progression-free survival in MIBC		
	HR	95% CI	*p* value	HR	95% CI	*p* value
*Univariate*						
Age (< 62 vs ≥ 62)	1.23	0.60–2.52	0.60	1.59	0.53–4.74	0.40
Gender (F vs M)	1.06	0.41–2.75	0.90	0.86	0.24–3.08	0.82
Tumor size (< 4 vs ≥ 4)	0.92	0.46–1.84	0.80	1.60	0.50–5.1	0.43
Grade (Low vs high)	0.34	0.16–0.74	0.006*	0.43	0.15–1.24	0.12
Tumor stage (early vs late)	0.38	0.18–0.77	0.007*	1.76	0.23–13.5	0.60
Smoking (nonsmokers vs smoker)	0.84	0.40–1.73	0.63	2.33	0.78–6.98	0.13
LN metastasis (No vs yes)	0.72	0.27–1.87	0.50	0.62	0.14–2.76	0.53
*RBBP8* (Low vs high expression)	4.1	1.89–8.97	< 0.001*	3.39	1.16–9.5	0.025*
*MSH4* (Low vs high expression)	4.99	2.45–10.2	< 0.001*	4.56	1.52–13.7	0.007*
*RBBP8* (UM vs M)	0.34	0.17–0.71	0.029*	0.29	0.10–0.86	0.0257*
*MSH4* (UM vs M)	0.43	0.21–0.89	0.023*	0.50	0.18–1.43	0.20
*Multivariate*						
Grade (Low vs high)	0.39	0.17–1.1	0.06			
Tumor stage (early vs late)	1.38	0.18–10.8	0.76			
*RBBP8* (Low vs high expression)	2.44	0.45–13.5	0.30	0.88	0.13–5.97	0.90
*MSH4* (Low vs high expression)	2.37	0.78–7.16	0.13	4.39	1.39–13.8	0.012*
*RBBP8* (UM vs M)	0.85	0.27–2.7	0.79	0.88	0.04–1.93	0.20
*MSH4* (UM vs M)	0.99	0.31–3.20	0.99			

*HR* hazard ratio, *LN* lymph node, *RBBP8* retinoblastoma binding protein 8, *UM* unmethylated, *M* methylated

^*^Significant at *p* < 0.05. Hazard ratio was calculated using COX regression survival analysis

## Discussion

In postgenomic era, it has been proposed global epigenetic aberrations maintained in carcinogenesis may play an important role in tumor heterogeneity (Sandoval and Esteller 2012). Promoter hypermethylation and mutational silencing of HR and MMR genes, such as *RB*, *BRCA1/2*, *PTEN*, *MLH1*, *MSH3*, *MSH6* have been identified in human cancer (Bhattacharya and Patel 2018; Hatziapostolou and Iliopoulos 2011).

In the present study, we aimed to identify differential methylation of DDR genes in UBC as compared to NC and in MIBC as compared to NMIBC. Hierarchical clustering of genome-wide methylome of TCGA−BLCA samples identified 12 out of the 154 DDR genes whose promoter region close to TSS to be hypermethylated. Then, we found that *RBBP8 and MSH4* were the most significant aberrantly methylated genes in TCGA−BLCA primary tumors compared to normal tissues. In silico analysis was validated by our MSP and qRT-PCR that detected a significant increase in *RBBP8* and *MSH4* hypermethylation and downregulation in UBC compared to NC. Interestingly, we found for the first time that *RBBP8* and *MSH4* methylation and their corresponding gene downregulation were significantly associated with progressive UBC which is in parallel with high tumor stage and muscle-invasive disease. This could not be assessed in the TCGA−BLCA datasets because most of TCGA cases were of the muscle-invasive subtype. In the present study, we demonstrated a significant positive correlation between *RBBP8* and *MSH4* methylation and reduced expression of their corresponding genes which suggests that *RBBP8* and *MSH4* hypermethylation account for their epigenetic inactivation in UBC. Moreover, correlation analysis have demonstrated a moderate positive between *RBBP8*- and *MSH4*- expression in UBC. These results indicate that MMR and HR methylation and gene inactivation are strongly correlated in UBC patients. This was not previously reported in UBC however, a previous study has shown a strong correlation between mutational status of HR and MMR genes that subsequently leads to genomic instability in gastric carcinoma patients (Liu et al. 2019). Hypermethylation of *RBBP8* has been previously detected as biomarker for bladder cancer patients (Mijnes et al. 2018). Microsatellite instability in urine has also been implicated as a noninvasive tool for diagnosis of BLCA (Zekri et al. 2019). Moreover, combined mutations in *MSH4* and *MLH3* were associated with increased risk of breast cancer (Conde et al. 2009). Collectively, this highlights the possible role of *RBBP8* and *MSH4* in BLCA susceptibility.

Despite current advances in surgical procedures and neoadjuvant chemotherapy, a large proportion of patients with NMIBC disease are at a high risk of progression to MIBC (Van Rhijn et al. 2009). In MIBC, patients are associated with increase in metastatic spread that eventually leads to less favorable outcome (Hautmann et al. 2006; Shariat et al. 2006). In recent decades, there has been a little progress in systematic chemotherapy for UBC (Sonpavde et al. 2016) except for a few including immunotherapeutic approaches (Inman et al. 2017). The first-line therapeutic approach of MIBC includes neoadjuvant-platinum-based combination chemotherapy or radiotherapy with or without concomitant systematic chemotherapy (Konety and Joslyn 2003; Poletajew et al. 2016). The delay of radical cystectomy in MIBC patients who do not respond to cisplatin is one of the drawbacks of neoadjuvant chemotherapy (Gore et al. 2009). Moreover, the lack of proper biomarkers that could predict muscle invasion and identify patients who will respond to cisplatin decreases the ability to select an appropriate treatment for BLCA (Bertz et al. 2014; Otto et al. 2011). Recently, epigenetic modifications have been implicated in modulation of treatment response in cancer (Lu et al. 2020).

In the current study, we aimed to assess epigenetic modification of *RBBP8* and *MSH4* as possible prognostic and predictive biomarkers for UBC patients (Lu et al. 2020). Our principal finding was the significant association of *RBBP8* and *MSH4*-methylation corresponding to gene inactivation with increase in response by 57.1% and 65.0%, respectively. In line of our results, loss of function mutations in DDR genes including *ATM*, *FANCC* and *ERCC2* have been associated with increased sensitivity to cisplatin-based chemotherapy and immunotherapies in MIBC (Abbosh and Plimack 2018). However, MMR status and chemosensitivity status has not been previously addressed in UBC.

*BRCA1* promoter methylation has been associated with favorable response to platinum-based treatment in breast and ovarian cancer (Stefansson et al. 2012) which is the standard curative approach in UBC management. DNA-damaging agent-like platinum-based chemotherapy elicit its effect through induction of inter-strand crosslink that leads to DNA DSB that are regularly repaired by HR and MMR (Rycenga and Long 2018). CtIP/RBBP8 protein is known to modulate the functions of *BRCA1* in transcriptional regulation and DNA repair. Thus, loss of function mutation of DDR will abolish cell’s ability to repair DSB and consequently resulting in o treatment susceptibility (Hoa et al. 2015; Makharashvili et al. 2014).

With current interest in precision medicine, we aimed to develop a predictive model for response to cisplatin-based therapy in UBC based on the *RBBP8* and *MSH4* methylation along with patients’ characteristics which might satisfy patients’ needs to more personalized medicine. We found that the best predictive model for stratification of patients according to response to therapy was a combination of KNN, RF and DT presenting an accuracy of 90.0%.

Survival analyses have also shown that that *RBBP8* and *MSH4* methylation and their corresponding gene downregulation significantly correlated with longer PFS in UBC patients. Moreover, *RBBP8* methylation along with *RBBP8* and *MSH4* mRNA expression were associated with longer PFS in MIBC patients. Based on the multivariate survival analysis, we found that *MSH4* downregulation was the sole independent predictor of PFS in MIBC patients.

Thus, it can be inferred that unrepaired double-stranded crosslinks because of loss of *RBBP8* and *MSH4* function may also increase cellular sensitivity to cisplatin-based chemotherapy and improve patients’ outcome especially in MIBC. In a recent study, deficiency of *RBBP8* expression has been implicated to increase sensitivity to cisplatin-based therapy in BLCA (Mijnes et al. 2018). Moreover, *RBBP8* deficiency has been associated with increase the susceptibility of breast and ovarian cancer to poly ADP ribose polymerase (PARP) inhibitors in a manner similar to *BRCA1* mutations (Lin et al. 2014; Wang et al. 2016) which suggests a possible benefit of difficult to manage MIBC from this approach.

To our knowledge, this is the first study to address a relationship between epigenetic inactivation of *MSH4* and response to cisplatin-based chemotherapy in UBC patients. However, inactivation of MMR genes like *MSH3* and *MSH5* by methylation and single nucleotide polymorphisms (SNPs) has been associated with increased sensitivity to cisplatin-based chemotherapy in small cell lung carcinoma (J.-Y. Liu et al. 2017).

In conclusion, our data showed that epigenetic inactivation of *RBBP8* and *MSH4* by hypermethylation was significantly frequent in MIBC subtype and significantly associated with favorable outcome in terms of PFS and increase sensitivity to cisplatin-based chemotherapy. Our machine-learning model revealed that *RBBP8* and *MSH4* methylation could provide a tool for prediction of UBC patients who might respond to platinum-based chemotherapy taking in consideration patients’ clinical data. This study should be expanded to multiple centers for further verification of the potential role of *RBBP8* and *MSH4* in UBC a step towards personalized medicine.

## Supplementary Information

Below is the link to the electronic supplementary material.

Supplementary file1 (TIF 7278 KB) Supplementary Fig. 1. The pipeline used to develop the machine-learning classification model for prediction of treatment response based on patients’ characteristics along with RBBP8 and MSH4 methylation.

Supplementary file2 (XLSX 15 KB)

Supplementary file3 (DOCX 15 KB)

Supplementary file4 (DOCX 14 KB)

## Data Availability

Data available upon request.
