# Supplementary figures and images for: Epigenetic inactivation of DNA repair genes as promising prognostic and predictive biomarkers in urothelial bladder carcinoma patients
Source: Mol Genet Genomics. 2022 Sep 8;297(6):1671–87. doi: 10.1007/s00438-022-01950-x (PMC9596572; doi:10.1007/s00438-022-01950-x)

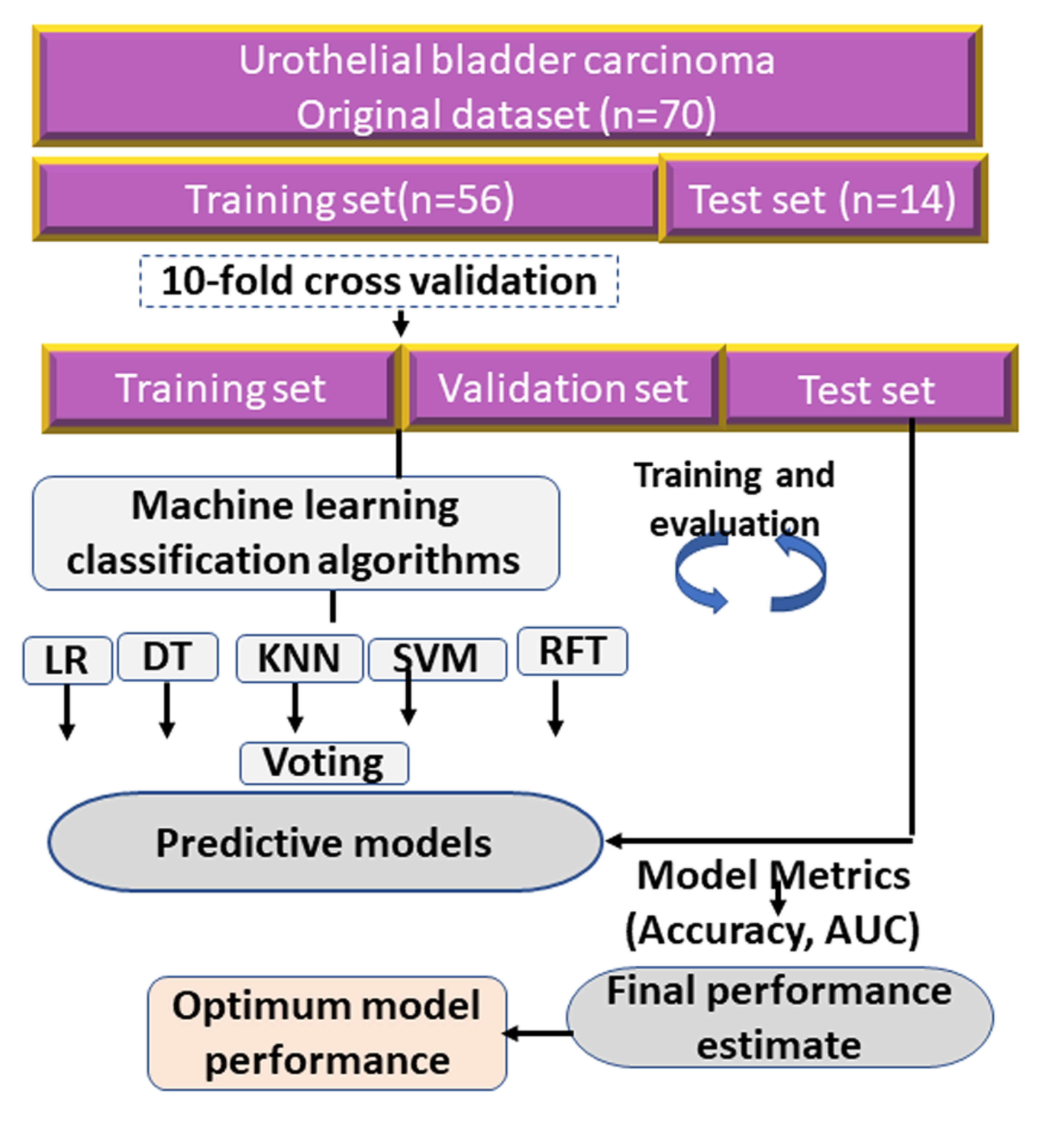

Supplement: Supplementary file 1 — Supplementary file1 (TIF 7278 KB) Supplementary Fig. 1. The pipeline used to develop the machine-learning classification model for prediction of treatment response based on patients’ characteristics along with RBBP8 and MSH4 methylation. [file 438_2022_1950_MOESM1_ESM.tif]
