# Supplementary material for: Epigenetic inactivation of DNA repair genes as promising prognostic and predictive biomarkers in urothelial bladder carcinoma patients
Source: Mol Genet Genomics. 2022 Sep 8;297(6):1671–87. doi: 10.1007/s00438-022-01950-x (PMC9596572; doi:10.1007/s00438-022-01950-x)
Supplement: Supplementary file 3 — Supplementary file3 (DOCX 15 KB) [file 438_2022_1950_MOESM3_ESM.docx]

**Table S2.** The sequence of oligonucleotide primers used for methylation specific PCR (MSP)

| **Gene** | **Sequence (5' → 3')** | **Annealing Temp. (ºC)** |
| --- | --- | --- |
| *RBBP8-*M | Forward: 5'-CGTTA GATTT TTCGG GTAGT TTTCG GTAGT TTC-3' | 60 |
|  | Reverse: 5'-CGTTA GATTT TTCGG GTAGT TTTCG GTAGT TTC-3' |  |
| *RBBP8-*U | Forward: 5'-TTGTT AGATT TTTTG GGTAG TTTTT GGTAG TTTTG-3' | 60 |
|  | Reverse: 5'-CATAC CCTAA CTAAA AAACA AACTC TTCAA TACA-3' |  |
| *MSH4-*M | Forward: 5’-GAGGG GTYGT TTAGA AATTT TAT-3’ | 60 |
|  | Reverse: 5’- CAAAA AAAAC TACTA CTAC TAC-3’ |  |
| *MSH4-*U | Forward: 5’-TTATG TGGAA AGTTG AGTTT GTTTG-3’ | 60 |
|  | Reverse: 5’-CCCAA TCACT CCTTA CCTTA AAC-3’ |  |

**MSP cycling conditions:** The PCR was initiated at 95°C for 2 min followed by 35 (for tissue)-38 cycles (for urine) cycles of 95°C for 15 s, 60°C for 20 s, 72°C for 20 s and a final extension at 72°C for 5 min. Primers designed by BiSearch Web Server (<http://bisearch.enzim.hu/>).
