# Supplementary material for: Epigenetic inactivation of DNA repair genes as promising prognostic and predictive biomarkers in urothelial bladder carcinoma patients
Source: Mol Genet Genomics. 2022 Sep 8;297(6):1671–87. doi: 10.1007/s00438-022-01950-x (PMC9596572; doi:10.1007/s00438-022-01950-x)
Supplement: Supplementary file 4 — Supplementary file4 (DOCX 14 KB) [file 438_2022_1950_MOESM4_ESM.docx]

**Table S3.** The sequence of oligonucleotide primers used for quantitative real time PCR (qRT-PCR)

| **Gene** | **Sequence (5' → 3')** | **Annealing Temp. (ºC)** |
| --- | --- | --- |
| *RBBP8* | Forward: 5'- CTTCA TTCGG TACAG GTCAC TC -3' | 60 |
|  | Reverse: 5'- GTCCC TTCTC CATCC ATTTC TC -3'- |  |
| *MSH4* | Forward: 5'- TGGAC ACCAC AAGTG GGATA-3' | 60 |
|  | Reverse: 5'- TTGCC ATTCC TATTT CACCTC -3' |  |
| *GAPDH* | Forward: 5’-GAAGGTGAAGGTCGGAGTCA-3’ | 60 |
|  | Reverse: 5’-TGGACTCCACGACGTACTCA-3’ |  |

**Cycling conditions for one-step qRT-PCR:** 55°C for 15 mins, 95°C for 5 mins, 95°C for 30 secs, followed by 40 cycles of 60°C for 30 secs. Primers designed by BiSearch Web Server (<http://bisearch.enzim.hu/>).
